# Intussusception in Young Children: Protocol for Multisite Hospital Sentinel Surveillance in India

**DOI:** 10.3390/mps1020011

**Published:** 2018-03-22

**Authors:** Manoja Kumar Das, Narendra Kumar Arora, Jan Bonhoeffer, Patrick L. F. Zuber, Christine G. Maure

**Affiliations:** 1 The INCLEN Trust International, New Delhi 110020, India; nkarora@inclentrust.org; 2 University Children’s Hospital (UKBB), University of Basel, 4056 Basel, Switzerland; contact@medarcadia.com; 3 Coordinator, Brighton Collaboration Foundation, 4056 Basel, Switzerland; 4 Department of Essential Medicines and Health Products, World Health Organization, 1211 Geneva 27, Switzerland; zuberp@who.int (P.L.F.Z.); maurec@who.int (C.G.M.)

**Keywords:** intussusception, retrospective, prospective, surveillance, rotavirus vaccine

## Abstract

India has recently introduced a rotavirus vaccine under a universal immunization program. There is limited information on intussusception, an adverse event, following immunization in children from India. We are conducting sentinel surveillance for intussusception in children aged under two years at 19 hospitals. The sentinel sites’ selection followed a multistage process. The surveillance combines retrospective surveillance for 69 months and prospective surveillance for 18 months. The suspected intussusception cases shall be reviewed for capturing confirmed cases and detailed data collection and classification according to Brighton Collaboration criteria. Data shall be analysed to describe epidemiology, trends, regional and seasonal variations, clinical profiles, management modalities, and outcomes of intussusception. The combination of prospective and retrospective surveillance shall be informative about the trend of intussusception over the last seven years in India. At four sites where rotavirus vaccines have been introduced, the change in intussusception trends shall be documented. The potential association with rotavirus vaccines and other vaccines shall be assessed using case-control and self-controlled case series methodology. Results are forthcoming. The results shall support the national vaccine safety surveillance effort by providing baseline estimates of intussusception for continued monitoring. The surveillance protocol and site selection processes shall inform similar vaccine-safety surveillance in India and other developing countries.

## 1. Introduction

Rotavirus is the most common cause of severe and dehydrating diarrhoea leading to hospitalisation and death among children aged under five years, globally [1]. Vaccination is the most effective intervention for preventing hospitalisation and death in children. However, Rotashield (Wyeth Lederle, Marietta, PA, USA), the first available rotavirus vaccine, was withdrawn in 1999 due to an increased frequency of intussusception in the first two weeks after vaccination [2]. Prelicensure clinical trials of subsequent candidate vaccines included over 60,000 participants to monitor the risk of intussusception [3,4]. While no increased risk was seen in some populations (USA [5] and Brazil [6]), a small rise in risk of intussusception (~1–2 excess cases per 100,000 vaccinated infants) was seen in others (Mexico [6] and Australia [7]). Based on the evidence of efficacy and safety outside India, both the candidates of RotaTeq (RV5, Merck & Co., Inc., Kenilworth, NJ, USA) and Rotarix (RV1, GlaxoSmithKline Biologicals, Rixensart, Belgium) are licensed and are being used in India. The licensed Indian vaccine, Rotavac (116E; Bharat Biotech, India) did not document an increased risk of intussusception in infants (*n* = 6799) [8,9]. In another recently licensed Indian rotavirus vaccine, Rotasiil (BRV-PV, Serum Institute of India, Pune, India), no increased risk of intussusception was documented (*n* = 7500) [10]. However, the number of recruited infants in both these phase three trials were inadequate to document the real risk of intussusception. Based on the recommendation of the National Technical Advisory Group on Immunization (NTAGI), the Government of India introduced the rotavirus vaccine Rotavac under the Universal Immunization Program (UIP) in a phased manner. Under the UIP, the first dose of rotavirus vaccine is being administered up to one year of age, allowing completion of the schedule in the second year [11]. The World Health Organization (WHO) recommends administration of rotavirus vaccines up to two years of age [12]. The aetiology of intussusception remains to be elucidated. It is the most common cause of bowel obstruction in infants and young children. A review on intussusception including 88 studies demonstrated a mean incidence of 74/100,000 infants (range: 9–328), with the peak among infants of five to seven months of age. The incidences in developed countries ranged from 20 to 101 per 100,000 infants. The incidences in developing countries from Asia, Africa, and Latin America varied widely, and were 9–328, 17–56, and 30–51 per 100,000 infants, respectively [13]. The wide use of the Brighton collaboration standardized case definition of intussusception has reduced misclassification and increased data comparability [14]. In north India, the incidence of intussusception requiring hospitalisation was 17.7 (range: 5.9–41.4) cases per 100,000 infants [15]. Hospital-based retrospective surveillance from India reported that the episodes of intussusception in infants ranges from five to 10 episodes annually [13,16,17,18]. The reasons for regional variation are unknown, and may reflect methodological differences in study design or real differences in risk factors. Therefore, more data from different regions in India is needed to establish a reliable baseline and identify potential risk factors based on harmonized methods and international standards.

## 2. Experimental Design

### 2.1. Objectives

This sentinel surveillance project was designed to generate information on intussusception epidemiology among children aged one month to two years in India, to inform the policy and program of rotavirus vaccines and future surveillance. The objectives were: (1) to establish a hospital-based surveillance system for intussusception in India, considering the sociocultural and geographic diversity; (2) to document the burden, trend, and epidemiology of intussusception over the past five years and nine months at the surveillance sites; (3) to document the trend and epidemiology of intussusception and potential linkage with rotavirus vaccines over 18 months at the surveillance sites.

### 2.2. Design

This study combined retrospective and prospective sentinel surveillance for intussusception at the selected sites.

## 3. Procedure 

### 3.1. Selection of Sites and Institutions

**Figure 1:**
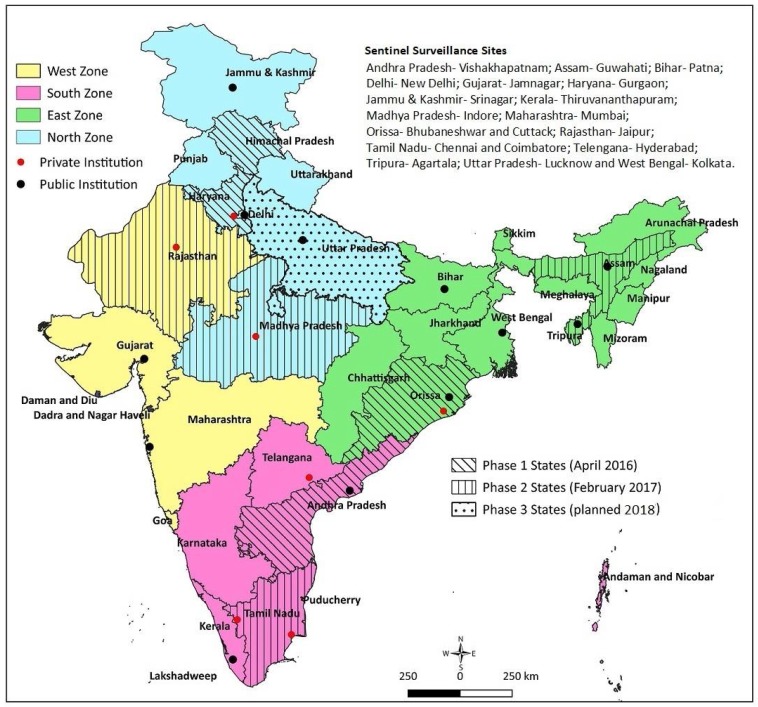
Surveillance network sites and rotavirus vaccine introduction in India.

Reliable sentinel surveillance requires the selection of appropriate institutions, particularly in the developing-country context. We predefined the following systematic processes: (1) Shortlisting: for regional representation, we shortlisted 40 potential institutions (10 per region) with appropriate facilities, including for pediatric surgery; (2) Screening: a preliminary telephone interaction with the concerned faculty at the potential institutions initiated the process. A structured questionnaire was sent to the 40 potential institutions, to obtain information on case load, clinical capacity in pediatrics and pediatric surgery, radiodiagnosis, pathology, availability of immunization source documents, and medical record-keeping system. The commitment of the site investigator, support from the department and institution, and time needed for administrative and ethical approval were also solicited. Based on the information received from the institutions and discussion with the faculty, 25 institutes were found to be eligible; (3) Site visits: the selected 25 institutions were visited by a technical expert. The technical expert verified the information provided in the screening questionnaire, and assessed the medical record-keeping practices and retrieval ability, immunization source document availability for the admitted children, investigator’s commitment, support from colleagues and the department, and the institute’s leadership to undertake the project. Based on the site visit feedback, 19 institutes (three to six institutions from each region) were included in the study (Figure 1).

### 3.2. Study Sites

The surveillance network involved 19 tertiary care hospitals, including medical college hospitals (both public and private institutions) and private hospitals. Three to six hospitals, including at least one private-sector hospital, represented the four regions (north, south, east, and west) of India, considering the geography and sociocultural diversity. Rotavirus vaccines have been introduced in nine states of India until now, in two phases: four in phase 1 (since April 2016) and five in phase 2 (since February 2017). Ten network institutions are located in these states; four institutions in phase 1 and six institutions in phase 2 states (as reflected in Figure 1).

### 3.3. Administrative and Regulatory Approvals

The necessary administrative and ethical approvals from all participating and coordinating institutions were obtained. The Ministry of Health and Family Welfare, Government of India, approved the study. The national immunization program manager was part of the technical advisory group (TAG) guiding the project.

### 3.4. Study Period

The surveillance was conducted in two components. The retrospective surveillance was conducted for 69 months (July 2010–March 2016) and the prospective surveillance was conducted for 18 months (April 2016–September 2017) at all the sites. Thus, the study, in total, collected data for 7 years and 3 months.

### 3.5. Case Identification and Enrollment

The hospitalised children aged >1 month and <24 months were included in the study. The eligible cases were identified adopting different processes for retrospective and prospective surveillance components: (1) Retrospective surveillance: we recognised that the institutions participating were following variable record-organisation methods; some following International statistical classification of diseases and related health problems (ICD) coding practice and some diagnosis-based classification [19]. At the institutions using ICD coding, both ICD-9 and ICD-10 systems were observed for the period of surveillance. For those institutions, in consultation with the technical experts, we identified eight clinical conditions with similar codes according to ICD-9 or ICD-10 systems, as specified in Table 1. For institutes not using ICD systems for classification, the diagnoses, including intussusception, acute intestinal obstruction, subacute intestinal obstruction, acute abdomen (including vomiting, painful abdomen, and abdominal distention), and blood in stool with vomiting, were used for identifying the suspected cases. The research team reviewed the medical records for all these suspected cases, either by an ICD system or diagnosis-based system. In a log of hospitalised cases in the eligible age group, case sheets retrieved and screened, suspected, and confirmed cases identified were maintained. (2) Prospective surveillance: for the prospective surveillance, the patients admitted with any of the diagnoses, including intussusception, acute intestinal obstruction, subacute intestinal obstruction, acute abdomen, painful abdomen (excessive crying), abdominal distension, and blood in stool with vomiting, were considered as suspected cases. All these suspected cases were tracked to identify the confirmed intussusception cases for recruitment. A log of cases screened, suspected cases tracked, and confirmed cases identified was maintained.

### 3.6. Data Collection

The data collection for retrospective and prospective components adopted the following steps: (1) Retrospective surveillance: (a) the list of suspected cases as per the ICD codes or final diagnoses were identified from the medical records department; (b) Additionally, we screened all registers from clinical (pediatrics, pediatric surgery, and emergency, if applicable) wards, operation theatres, and radiology (ultrasound and barium) and pathology departments to match the cases identified and identify any missed cases. A seal was used to mark completion of screening of the registers, with the date of screening and signature of the research staff; (c) The case sheets of all suspected cases were reviewed in detail to identify the confirmed intussusception cases; (d) For the confirmed intussusception cases, the data including clinical features, management/intervention, and final outcome were extracted into the case record forms (CRFs). The case screening process is documented in two log sheets: log sheet 1 (for documenting the suspected cases identified from the medical records department) and log sheet 2 (for the cases identified from supplementary sources such as registers from clinical wards, operation theatres, and radiology and pathology departments). The suspected and confirmed intussusception cases were verified from the medical case records; (2) Prospective surveillance: (a) all hospitalisations in the eligible age group were screened for identifying the suspected cases as per the criteria; (b) The suspected cases were tracked daily until final diagnosis; (c) All confirmed intussusception cases were approached for informed consent from a parent or legally authorised representative. A standardised study information sheet and informed consent form in the preferred local language was used for obtaining consent. For illiterate parents, information was communicated in the presence of an impartial literate witness for obtaining consent; (d) Following recruitment, the desired data on clinical features, course of events, management/intervention, and outcome; sociodemography; feeding; and immunization exposure were collected into the CRFs. The immunization exposures for all vaccines were collected from a reliable source (immunization card or immunization register at the facility). The processes of screening of cases, obtaining consent, and recruitment were documented in log sheet 3 on a daily basis; (3) Documentation: to ensure documentation clarity and minimal confusion, differently coloured CRFs (yellow for retrospective and green for prospective surveillance) and log sheets for the retrospective and prospective components were used.

### 3.7. Site Research Teams

At each surveillance site, the lead site investigator supervised the data collection and coordinated with a multidisciplinary team of faculty from pediatrics, surgery or pediatric surgery, radiology, pathology, and medical records. The site teams (investigators and research staff) received training on surveillance methods and data extraction following a standard training protocol facilitated by the central coodinating unit (CCU) and TAG experts. The clinical teams (doctors, faculty and residents, and nurses) in pediatric surgery and pediatric and radiology departments at each site hospital were orientated to enable smooth and efficient surveillance.

### 3.8. Case Adjudication and Labeling

A case adjudication committee (CAC) comprised of a pediatrician, pediatric surgeon, and radiologist are reviewing the CRFs of intussusception cases and relevant supporting documents to assign the diagnostic certainty level, according to Brighton Collaboration criteria [14].

### 3.9. Quality Assurance

Multilevel quality assurance and data quality-checking processes were put in place to ascertain protocol adherence and level of rigor and completion of surveillance for both retrospective and prospective components. Each site was visited by the TAG experts for assessing the processes for retrospective and prospective surveillance. The components assessed and verified for the retrospective surveillance methodology included: (a) the process of record retrieval and record completeness; (b) identification of the suspected cases from various sources; and (c) verification of the quality of data extraction for the confirmed intussusception cases. The components assessed and verified for prospective surveillance included: (a) daily surveillance and identification of suspected cases; (b) tracking of the suspected cases and confirmation of diagnosis; (c) obtaining consent; and (d) quality of data extraction in the CRFs. For randomly identified case sheets (for both retrospective and prospective components), the data in the CRFs were matched to assess their completeness and quality. A member from the CCU data team visited the study sites and verified the admissions for the years 2015, 2016, and 2017 (until September) from the medical records section, to identify any missed cases. The CCU member also verified data collected for all the confirmed intussusception cases. The CRFs received from the sites were reviewed by the CCU data team. Any data-related query is clarified with the site teams electronically by review of the source documents.

### 3.10. Data Management

Each CRF at the surveillance sites was given a unique identify number. Double data entry is done for the CRFs received from the sites using a customised data entry platform with an inbuilt data matching system. The matched and verified data is archived in the server with authorised access and regular backup.

### 3.11. Project Management

The study is managed by the principal investigator, supported by the research team, and guided by a multidisciplinary TAG with experts from vaccinology, paediatrics, paediatric gastroenterology, paediatric surgery, public health, and program and medical record management. A monthly call with the site teams (coordinator and research staff) is being done to track the progress at sites and resolve challenges.

### 3.12. Ethical Aspects

The study is being conducted in accordance with the Declaration of Helsinki, and the protocol has been approved by the Ethics Committee of INCLEN (protocol reference IIEC 023). For the prospective surveillance, all the subjects (a parent or legally authorised representative) gave their informed consent for inclusion before they participated in the study. For the retrospective surveillance, data have been collected from hospital case records in anonymity, without any subject identified, and exempted by ethics committees.

### 3.13. Linkage with the Public Health Surveillance

The surveillance system is linked with the surveillance of vaccine safety/adverse events following immunization (AEFI) under the universal immunization system. The intussusception cases with rotavirus vaccine exposure within four weeks shall be shared with the AEFI secretariat.

### 3.14. Dissemination

The findings shall be shared with the Indian national government and state program managers in immunization, vaccine safety, and public health surveillance. The findings shall be shared with the academic and research community through peer-reviewed journals and meetings.

## 4. Expected Results

The demographic and clinical characteristics of case-patients from all sites will be summarized using descriptive statistics. To document the seasonal variation and age distribution of the intussusception cases, the number of intussusception hospitalisations at the site, region, and national levels will be plotted by year and week of age. The trends over time in the number of intussusception cases and rate per 1000 pediatric hospitalisations shall be examined from July 2010 through to September 2017. The clinical features, site of intussusception, modality of diagnosis, treatment- and outcome-related characteristics, and level of certainty according to Brighton Collaboration criteria will be summarized and compared across the sites and regions. The agreement between clinical diagnosis and different levels of diagnostic certainty according to Brighton Collaboration criteria shall be estimated using the kappa statistic. The interval for admission will be compared with the intervention and outcome for the sites and regions. Coadministration of rotavirus vaccines within seven and seven to 21 days and other UIP vaccines within four weeks for the intussusception cases will be reported. To document any change in the number of intussusception cases before and after the introduction of vaccines, the number of intussusception cases per year will be plotted for the sites with rotavirus vaccines in the UIP during the period of 2016–2017. All statistical analyses will be performed using STATA-15 (StataCorp LLC, College Station, TX, USA) and Microsoft Excel (Microsoft^®^, Redmond, WA, USA).

## 5. Discussion

Intussusception has been a concern associated with rotavirus vaccines for some time. A meta-analysis reported a significant rise in the risk of intussusception after rotavirus vaccination if the first dose was administered after three months of age [20]. India recently introduced rotavirus vaccines (Rotavac and Rotasiil), as part of the UIP, in a phased manner. WHO advises the countries introducing new vaccines to establish active vaccine safety surveillance [21,22,23]. Review of the literature on intussusception epidemiology identified variable surveillance methodology used by studies across the globe, and ICD codes used were not mentioned [13]. The WHO protocol on the postmarketing surveillance of rotavirus vaccines does not mention the clinical conditions or ICD codes to be used for surveillance [23]. The protocol presented in this paper is developed for the first multisite sentinel surveillance (combining retrospective and prospective) of intussusception in children aged under two years in India. The WHO Global Advisory Committee on Vaccine Safety (GACVS) has recommended a standardized approach specifically for the postmarketing surveillance of rotavirus vaccines [23]. However, the protocol needs adaptation of the surveillance process according to the local setting, existing vaccine safety surveillance, and pharmacovigilance program. The specific operational guideline for rotavirus vaccine introduction in India categorizes the features of intussusception as early- and late-stage [11]. Intussusception is a less-frequent clinical condition, and the clinical ability of the community and peripheral health facility level is limited for early detection. To enable early detection and referral of the potential intussusception cases, the sensitive clinical features need to be identified. The combined retrospective and prospective sentinel surveillance representing different regions will provide information on burden, epidemiology, regional and seasonal variation, and outcomes prior to vaccine introduction, and changes, if any, following vaccine introduction. Use of uniform protocols, surveillance rigor, and quality assurance measures will promote comparability of data from these institutions.

This study has several limitations. The sentinel surveillance sites have no defined catchment area. Thus, the population level incidence of intussusception cannot be estimated. Past case record availability and quality of documentation may pose challenges. As part of the vaccine safety surveillance effort, there is need for the generation of national evidence on the intussusception burden and change after rotavirus vaccine introduction.

## 6. Conclusions

The data collected through this sentinel surveillance shall inform the following on intussusception in India: (1) description of the epidemiology, trend over the years, and geographic and seasonal variation of intussusception among children aged under two years; (2) clinical features, management, and outcome of intussusception at the sites; (3) agreement between clinical diagnosis and level of certainty according to Brighton Collaboration criteria; and (4) the relationship between intussusception and rotavirus and other UIP vaccines; (5) For some surveillance sites, the trend of intussusception before and after rotavirus vaccine introduction under the national immunization program shall be available. The current study findings are expected to generate an information base to support the national immunization program and vaccine surveillance, and also inform the future active surveillance effort in India. The authors also hope to encourage other low- and middle-income countries to consider similar approaches. The data being collected under the project shall be made available on request on completion of the project.

## Figures and Tables

**Table 1:** Suspected cases for screening as per International Classification of Diseases (ICD) coding.

Clinical Conditions Considered as Suspected Cases	Codes	
	ICD 10	ICD 9
Intussusception	K56.1	560.0
Volvulus	K56.2	560.2
Gallstone ileus	K56.3	560.31
Other impaction of intestine	K56.4	560.30
Intestinal adhesions with obstruction	K56.5	560.81
Other and unspecified intestinal obstruction	K56.6	560.9
Ileus, unspecified	K56.7	560.1
Paralytic ileus	K56.0	
